# ENHANCED SUPPORT FOR CHINESE AND KOREAN AMERICAN DEMENTIA CAREGIVERS: INSIGHT FROM SERVICE PROVIDERS

**DOI:** 10.1093/geroni/igad104.3731

**Published:** 2023-12-21

**Authors:** Jing Wang, Min Kyoung Johnson, I tek Leong, Yaolin Pei, Mary Mittelman, Cynthia Epstein, Kyung Hee Lee, Bei Wu

**Affiliations:** University of New Hampshire, Durham, New Hampshire, United States; New York University, New York City, New York, United States; New York University, New York City, New York, United States; New York University, New York City, New York, United States; New York University, New York City, New York, United States; New York University, New York City, New York, United States; Yonsei University, Seoul, Republic of Korea; New York University, New York City, New York, United States

## Abstract

As members of two large Asian immigrant populations in the U.S., Korean and Chinese American family caregivers of persons with dementia (PWD) have unique challenges impacted by their cultural background. Many of them prefer to and are relying on social services providers who speak the same language as they do for support and assistance. As part of the project to inform the cultural adaptation of NYU Caregiver Intervention-Enhanced Support (NYUCI-ES) program for Chinese and Korean American caregivers with multiple chronic conditions, we explored the perspectives of 13 Chinese and 12 Korean services providers (physicians and social service staff). During focus group discussions, services providers shared their perspectives on care challenges facing these populations, including challenges in initiating advanced care planning, family conflict in decision making and caregiving, insufficient community resources, staffing shortage associated with the pandemic and some care aides’ stigmatic beliefs about providing dementia care. We identified nuances in the following aspects: utilization of home health aides’ help, attitudes towards diagnosis and education, approaches to selfcare, preferences for engagement settings, trust-building, and family involvement. For instance, Korean services providers highlighted Korean caregivers’ strong preference for home health aides of the same gender as PWD and having individual rather than group session to learn about dementia. What we found from services providers largely echoed our findings from family caregivers but also provided unique insights from their work experiences. These findings will inform cultural adaptation of the content of the NYUCI to improve its applicability to Chinese and Korean American caregivers.

